# SGLT-2 Inhibitors Versus GLP-1 Receptor Agonists in Patients with Type 2 Diabetes Mellitus: A Propensity Score-Matched Cohort Study Comparing Adherence and Persistence with Specific Reference to Cardiovascular Disease

**DOI:** 10.3390/ph19101607

**Published:** 2026-10-10

**Authors:** M. Asunción González-González, M. Aránzazu Pedrosa-Naudín, Diego Fernández-Lázaro, Isabel Díaz Planelles, F. Javier Álvarez, Eduardo Gutiérrez-Abejón

**Affiliations:** 1 Pharmacy Directorate, Castilla y León Health Council, 47007 Valladolid, Spain; asungonzlez@saludcastillayleon.es (M.A.G.-G.); maranzazup@saludcastillayleon.es (M.A.P.-N.); 2 Area of Histology and Neurobiology Research Group, Faculty of Medicine, University of Valladolid, 47003 Valladolid, Spain; diego.fernandez.lazaro@uva.es; 3 Facultad de Empresa y Comunicación, Universidad Internacional de la Rioja (UNIR), 26004 Logroño, Spain; isabel.diazplanelles@unir.net; 4 Area of Pharmacology, Faculty of Medicine, University of Valladolid, 47003 Valladolid, Spain; alvarez@uva.es; 5 CEIm of Valladolid Health Areas, Hospital Clínico Universitario de Valladolid, 47003 Valladolid, Spain; 6 Laboratory of Pharmacoepidemiological Research in Primary Care, Valladolid Health Research Institute (IBioVALL), 47010 Valladolid, Spain; 7 Atención Primaria, Área de Salud de Valladolid Este, 47010 Valladolid, Spain

**Keywords:** adherence, persistence, drug utilization, type 2 diabetes mellitus, cardiovascular disease, SGLT-2 inhibitors, GLP-1 receptor agonists

## Abstract

**Background/Objectives**: Poor adherence and persistence to sodium-glucose cotransporter-2 inhibitors (SGLT-2is) and glucagon-like peptide-1 receptor agonists (GLP-1 RAs) are a critical issue in the treatment of type 2 diabetes mellitus (T2DM), especially in patients with cardiovascular disease (CVD). We aimed to compare adherence and persistence to these drugs in routine care. **Methods**: A retrospective propensity score-matched (PSM) cohort study was conducted using pharmacy claims data. Patients aged 40 years or older with T2DM initiating SGLT-2is or GLP-1 RAs from 1 January 2019 to 31 December 2024 were included. Adherence was measured using the Medication Possession Ratio (MPR), and persistence was defined as the time to discontinuation (gap ≥ 60 days between refills). **Results**: At the 1-year follow-up, the SGLT-2i cohort showed significantly higher adherence compared to the GLP-1 RA cohort (86.66% vs. 76.53%; OR = 2.02) and higher persistence (71.48% vs. 66.52% at 12 months; HR = 0.83). The main factors associated with lack of persistency were newly diagnosed T2DM (HR = 1.25) and insulin use (HR = 1.32). In the CVD subgroup, the differences in favor of the SGLT-2i cohort were more pronounced for adherence (89.57% vs. 78.12%; OR = 2.44) and persistence (73.9% vs. 63.59%; HR = 0.66). **Conclusions**: In real-world clinical practice, adherence and persistence rates were higher in patients on SGLT-2is compared to those on GLP-1 RAs, particularly in the CVD subgroup. Our findings suggest that the new strategies are necessary to improve adherence and persistence to these medications, particularly in patients with CVD.

## 1. Introduction

Type 2 diabetes mellitus (T2DM) affects 8.5% of the population in Europe and the United States [1] and is estimated to affect 629 million people worldwide by 2045 [2]. T2DM is the ninth leading cause of mortality, accounting for more than one million deaths annually [1]. The economic burden of T2DM is also considerable, accounting for 10% to 15% of total healthcare expenses in developed countries [3].

T2DM is a chronic disease characterized by persistent hyperglycemia due to decreased insulin secretion and insulin resistance [4]. Chronic hyperglycemia contributes to endothelial dysfunction, oxidative stress, systemic inflammation, and atherogenic dyslipidemia [5]. In addition, insulin resistance is a contributing factor to atheroma plaque formation, ventricular hypertrophy, and diastolic abnormality [6]. Therefore, T2DM is a major risk factor for the development of cardiovascular disease (CVD), including ischemic heart disease (IHD), heart failure (HF), stroke, and peripheral artery disease [7]. Specifically, the risk of HF in patients with T2DM is twice that of the general population, and the risk of myocardial infarction and stroke is 4-to-6-fold higher [8]. Having both T2DM and HF or atrial fibrillation (AF) increases the risk of major adverse cardiovascular events (MACEs), hospitalizations, and mortality [9].

In addition to their glucose-lowering effects, two classes of oral antidiabetic medications (OADs) have been associated with a lower risk of cardiovascular events and mortality: sodium-glucose cotransporter-2 inhibitors (SGLT-2is) and glucagon-like peptide-1 receptor agonists (GLP-1 RAs) [10,11,12,13]. Accordingly, the European Association for the Study of Diabetes (EASD) and the American Diabetes Association (ADA) recommend using SGLT-2is and GLP-1 RAs for T2DM patients with established CVD, high risk of developing CVD, and established CKD, and/or HF, independent of glycemic control or concomitant metformin use [14].

As with other chronic diseases, a lack of medication adherence is a barrier to optimal T2DM control. Non-adherence to OADs has been associated with higher HbA1c levels, diastolic blood pressure, and LDL-cholesterol levels [15], with an increased risk of CVD events and mortality [16]. The following factors have been observed to be associated with non-adherence to OADs: perception of medication inefficacy, side effects, fear of hypoglycemia, lack of diabetes education, complexity of treatment, cost, and lack of medication access [17,18].

A meta-analysis of real-world data showed adherence rates ranging from 28% to 73% for SGLT-2is and from 20% to 74% for GLP-1 RAs [19]. These low adherence rates may be a therapeutic issue, since adherence to SGLT-2is and GLP-1 RAs is associated with a lower likelihood of starting insulin therapy, fewer hospitalizations, and lower all-cause mortality [20].

Despite the available evidence, direct comparisons between SGLT-2is and GLP-1 RAs in terms of adherence and persistence in the subgroup of patients with T2DM and CVD remain understudied, especially in the context of the Spanish public health care system.

Given these precedents, we evaluated real-world medication adherence and persistence in patients initiating SGLT-2is vs. GLP-1 RAs. We focused on adults with T2DM treated under routine care conditions in a public health care system in a large region of Spain. Additionally, patients with T2DM and CVD were analyzed separately. To ensure homogeneity of the population, we used propensity score-matched (PSM) cohorts.

## 2. Results

### 2.1. Study Cohort

**Figure 1:**
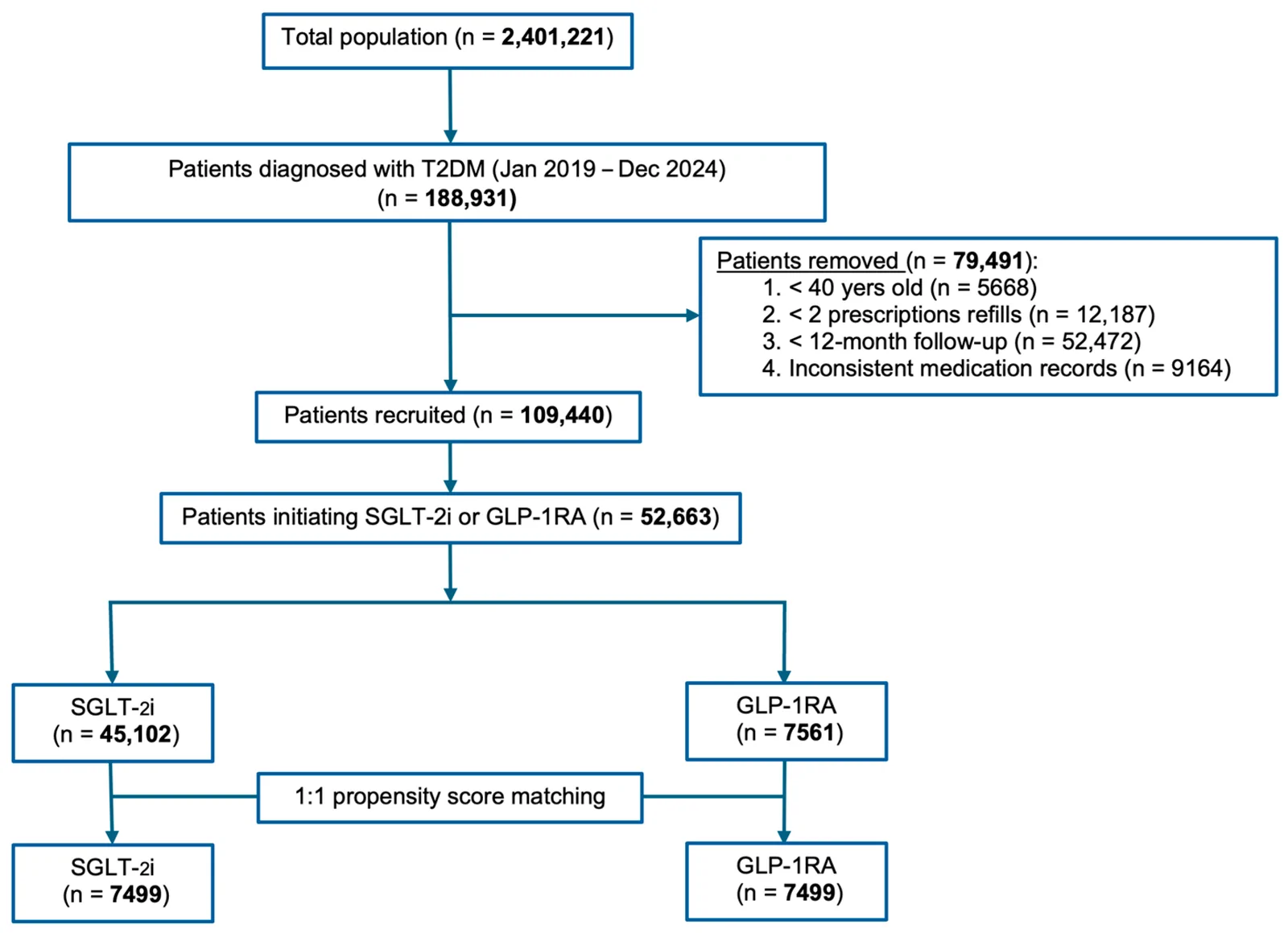
Flowchart of the study population (T2DM, type 2 diabetes mellitus; SGLT-2is, sodium-glucose cotransporter-2 inhibitors; GLP-1 RAs, glucagon-like peptide-1 receptor agonists).

We identified 52,663 patients with T2DM aged 40 years or older who initiated treatment with SGLT-2is or GLP-1 RAs. After 1:1 PSM, the SGLT-2i and GLP-1 RA cohorts each comprised 7499 patients (Figure 1). Baseline characteristics were well balanced, with all standardized mean differences (SMDs) < 0.1 (Table 1).

### 2.2. Baseline Characteristics

After matching, the cohort included 14,998 patients, of whom 7684 (51.23%) were male, with a mean age of 65.9 ± 10.84 years. Overall, 27.16% of patients were newly diagnosed with T2DM, with dapagliflozin and oral semaglutide accounting for most SGLT-2i and GLP-1 RA dispensations, respectively (Supplementary Table S1). Patients used insulin (40.9%) and other OADs (94.57%), particularly metformin (66.04%) and dipeptidyl peptidase-4 inhibitors (DPP-4is) (22.84%). The most common comorbidities were hypertension (64.48%), hyperlipidemia (60.99%), and chronic respiratory disease (25.52%), while CVD accounted for 22.02% (Table 1).

### 2.3. Adherence and Persistence

After 1:1 PSM, the SGLT-2i cohort reported a higher adherence prevalence than the GLP-1 RA cohort (86.66% vs. 76.53%; *p* = 0.001). Furthermore, patients in the SGLT-2i cohort were more likely to be adherent than those in the GLP-1 RA cohort (odds ratio (OR) = 2.02; 95% CI = 1.86–2.21) (Table 2). The results from the original cohort can be consulted in Supplementary Table S2.

**Figure 2:**
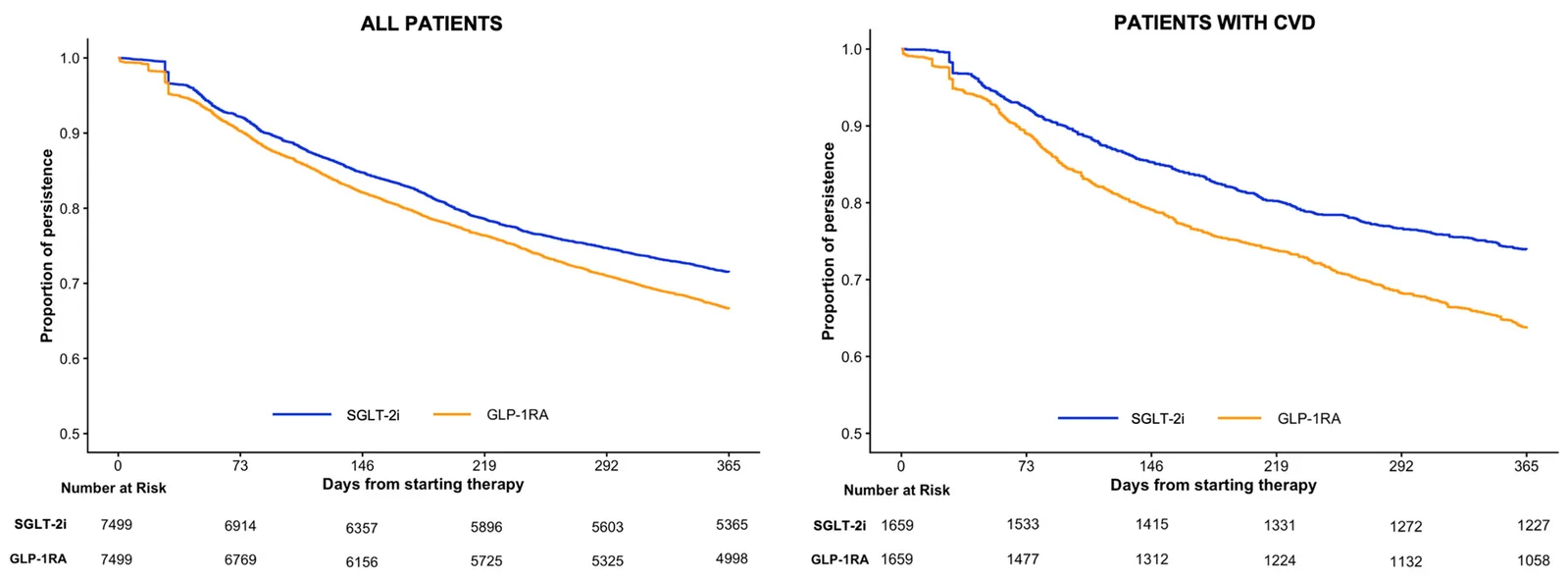
Kaplan–Meier analysis for treatment persistence in patients with T2DM (all patients and patients with cardiovascular disease (CVD)) on sodium-glucose cotransporter-2 inhibitors (SGLT-2is) and glucagon-like peptide-1 receptor agonists (GLP-1 RAs).

Persistence was higher in the SGLT-2i cohort than in the GLP-1 RA cohort at 3 (89.66% vs. 87.93%; *p* = 0.001), 6 (82.28% vs. 79.31%; *p* = 0.001), and 12 months (71.48% vs. 66.52%; *p* = 0.001). Also, mean treatment persistence was higher in patients in the SGLT-2i group than those in the GLP-1 RA group (303.76; 95% CI = 301.28–306.25 vs. 294.22; 95% CI = 291.58–296.85); *p* = 0.001). At the 12-month follow-up, patients in the SGLT-2i cohort are more likely to be persistent (hazard ratio (HR) = 0.83; 95% CI = 0.78–0.88) (Table 2, Figure 2). The results were comparable when the gap between prescription refills was modified from 60 to 90 days (HR: 0.84; 95% CI = 0.8–0.9).

After PSM, higher persistence was observed for male (HR = 0.9; 95% CI = 0.85–0.96), annual income ranging from €18,000 to €100,000 (HR = 0.65; 95% CI = 0.47–0.90), monotherapy for T2DM (HR = 0.81; 95% CI = 0.70–0.92), and hyperlipidemia (HR = 0.89; 95% CI = 0.84–0.94). Conversely, lower persistence was observed for newly diagnosed T2DM (HR = 1.25; 95% CI = 1.16–1.33), insulin use (HR = 1.32; 95% CI = 1.23–1.39), chronic respiratory disease (HR = 1.08; 95% CI = 1.01–1.15), anxiety (HR = 1.12; 95% CI = 1.04–1.2), and obesity (HR = 1.18; 95% CI = 1.01–1.37) (Table 3).

The global Schoenfeld residuals test yielded a *p*-value = 0.117, confirming the Cox proportional hazards assumption. All covariates satisfied the proportional hazards assumption, with *p*-values > 0.05. As shown in the Cox–Snell residual plot (Supplementary Figure S1a), the final model exhibited an optimal fit.

### 2.4. Subgroup Analysis

Among the subgroup of patients with concomitant CVD, after 1:1 PSM, 1659 patients were identified in the SGLT-2i cohort and 1659 patients in the GLP-1 RA cohort. The SGLT-2i cohort showed a higher prevalence of adherent and persistent patients than the GLP-1 RA cohort (89.57% vs. 78.12% and 73.9% vs. 63.59%, respectively). In addition, mean treatment persistence was higher in the SGLT-2i cohort than in the GLP-1 RA cohort (307.8; 95% CI = 302.67–313.08 vs. 285.94; 95% CI = 280.07–291.81; *p* = 0.001). Patients in the SGLT-2i group were more likely to be adherent and persistent than those in the GLP-1 RA group (OR = 2.44; 95% CI = 2.01–2.97 and HR = 0.66; 95% CI = 0.58–0.75) (Table 4, Figure 2).

Table 4 presents the adjusted results categorized by type of CVD. In all cases, adherence and persistence rates were higher in the SGLT-2i cohort than in the GLP-1 RA cohort. The greatest difference in favor of the SGLT-2i cohort was observed in patients with AF for adherence (OR = 2.64; 95% CI = 1.91–3.65) and in patients with IS for persistence (HR = 0.59; 95% CI = 0.41–0.83) (Supplementary Figure S2).

Among this subgroup, newly diagnosed T2DM (HR = 1.2; 95% CI = 1.04–1.54), insulin use (HR = 1.18; 95% CI = 1.03–1.35), chronic respiratory disease (HR = 1.14; 95% CI = 1.02–1.3), and anxiety (HR = 1.19; 95% CI = 1.02–1.41) have been shown to be factors associated with lack of persistence. As for the type of cardiovascular disease, only patients with AF were more likely to be adherent (OR = 1.46; 95% CI = 1.21–1.61) and persistent (HR = 0.88; 95% CI = 0.79–0.98) (Supplementary Table S3).

The global Schoenfeld residuals test yielded a *p*-value = 0.196, confirming the Cox proportional hazards assumption. All covariates satisfied the proportional hazards assumption, with *p*-values > 0.05. As shown in the Cox–Snell residual plot (Supplementary Figure S1b), the final model exhibited an optimal fit.

## 3. Discussion

In this propensity score-matched study, we present data comparing real-world medication adherence and persistence in Spanish patients with T2DM who initiated SGLT-2i or GLP-1 RA treatment between 2019 and 2024. After matching, 14,998 new users of SGLT-2is and GLP-1 RAs were identified, with 1 in 3 patients having newly diagnosed T2DM. Our results showed that approximately 80% of the patients were adherent, and 70% were persistent at the one-year follow-up. Moreover, patients who initiated treatment with SGLT-2is showed higher adherence (+10%) and persistence (+5%) than those who initiated treatment with GLP-1 RAs. These differences were more pronounced in the subgroup of patients with CVD. Patients with newly diagnosed T2DM using insulin and with comorbidities, such as chronic respiratory disease, anxiety, and obesity, were at higher risk of lack of persistence.

In our original cohort, we observed higher use of SGLT-2is compared to GLP-1 RAs. The use of GLP-1 RAs during the study’s follow-up period has been affected by the global shortage of semaglutide injectables between 2022 and 2025 due to increased demand for obesity treatment [21,22]. Conversely, oral semaglutide was approved in Europe in April 2020 [23] and accounted for approximately 50% of GLP-1 RA use in our study. However, as in other studies [24,25], we observed that the use of SGLT-2is and GLP-1 RAs is low, particularly in patients with CVD.

The adherence rate was higher among patients using SGLT-2is compared to those using GLP-1 RAs, as observed in other American [3] and European studies [26,27]. However, another European study [28] observed the inverse trend. Adherence rates range from 65% [27] to 73% [3] for the SGLT-2i group and from 61% [27] to 65% [3] for the GLP-1 RA group. Our results showed higher adherence values in both cohorts, although our measure of adherence is based on MPR, whereas other studies used the proportion of days covered (PDC) [3,27]. Therefore, comparisons should be made with caution, since PDC is a more conservative measure of adherence than MPR [29].

Our findings showed a higher proportion of persistent patients in the SGLT-2i cohort than in the GLP-1 RA cohort. These results are like those in other American [30] and European [26,27] studies but diverge from those in other studies [28,31,32]. As in other studies [28,31,32], the persistence rate in the SGLT-2i cohort exceeded 70%, while in other studies it was lower, ranging from 57% to 68% [26,27,30]. Furthermore, a meta-analysis showed persistence rates of 80%, 62%, and 46% at 6, 12, and 24 months of treatment, respectively [33]. In the GLP-1 RA cohort, the persistence rate was approximately 66%, like that reported in other studies [26,27]. However, other studies showed higher values ranging from 69% to 85% [28,31,34,35] and lower values around 50% [30]. Indeed, the results of published studies are heterogeneous and may be significantly influenced by the methodology used. In these studies, different definitions of persistence have been used, with gaps between prescription refills of ≥45 [34], ≥60 [27,31], ≥90 [28,32,35], and ≥180 days [32]. Studies with longer follow-up periods [27,28,34] have shown a decrease in the risk of discontinuation over time [28]. Additionally, our study includes all available GLP-1 RAs, unlike others that do not include liraglutide [35], semaglutide [31,34], or oral semaglutide [28,35,36]. This is relevant because significant differences in persistence rates have been observed among GLP-1 RAs, ranging from 69% for exenatide to 85% for dulaglutide [34]. The published studies cover the period from 2006 to 2022, whereas our study is more recent (2019–2024); therefore, persistence in the GLP-1 RA cohort may be influenced by the shortage of semaglutide injectables [21,22]. This scenario could have encouraged switching to other GLP-1 RAs or other OADs [37]. Moreover, switching between the injectable and oral formulations of semaglutide is complex, as the oral formulation displays higher pharmacokinetic variability in absorption compared to the injectable formulation [37,38]. According to a recent European study [39], injectable semaglutide was associated with higher persistence than oral semaglutide. The specific administration requirements [38] and gastrointestinal side effects associated with oral semaglutide [39] may contribute to non-use of the medication [40]. This scenario may have affected our findings, since in the GLP-1 RA cohort, the use of oral semaglutide was preferred. However, according to the REVISE study, patient preference between a drug with a once-daily oral formulation and a drug with a once-weekly injectable formulation is equivalent [41]. In addition, for injectable formulations, patients prefer a weekly dosing regimen over a daily one because it results in fewer missed doses [42]. No substantial differences have been observed among SGLT-2is [32], although some studies show a higher persistence rate for empagliflozin than for dapagliflozin [43].

In previous studies with other medications [44], our group observed higher therapeutic compliance among cardiovascular patients. In this study, among the subgroup of patients with CVD, higher rates of adherence and persistence were observed in the SGLT-2i cohort. On the other hand, although the GLP-1 RA cohort had a higher adherence rate, the difference in favor of the SGLT-2i cohort increased. Additionally, the persistence rate decreased in the GLP-1 RA cohort, and patients discontinued therapy earlier. This finding is consistent with other studies that found CVD to be associated with higher persistence to SGLT-2is, but not to GLP-1 RAs [45]. Specifically, HF or AF protects against treatment discontinuation in patients on SGLT-2is. Furthermore, IHD, HF, and stroke increase the likelihood of treatment discontinuation in patients on GLP-1 RAs [28]. However, our study directly compared cohorts, whereas other studies were conducted separately [28]. Therefore, a cautious interpretation is required. Furthermore, long-term studies are needed, as one study found that one-quarter of patients who discontinued therapy with SGLT-2is or GLP-1 RAs reinitiated therapy soon after [28].

As has been observed in other studies of chronic diseases [46,47], including T2DM [27], being male was associated with a higher likelihood of persistence. A higher annual income is associated with a lower risk of discontinuation [35,48], although this association is more evident in American studies [49] than in European studies [28]. In our study, a higher socioeconomic level had a positive influence on persistence. However, this effect was only significant among patients with annual incomes between €18,000 and €100,000. One possible explanation is that the population with an annual income exceeding €100,000 in our region is relatively limited. Another factor contributing to persistence is the treatment of T2DM with monotherapy, which is consistent with the findings of a meta-analysis [50].

Conversely, patients with newly diagnosed T2DM showed a 20% decrease in persistence. This finding is noteworthy, as it has been associated with an increased risk of macrovascular events, hospitalizations, and long-term mortality [51]. Another important predictor of decreased persistence was insulin use (24%). This finding is consistent with a meta-analysis that showed increased insulin use among non-persistent patients [33]. Comorbidities such as chronic respiratory disease, anxiety, and obesity were associated with a lack of persistence. As in our study, Malik et al. [28] observed a higher risk of discontinuation in patients with chronic obstructive pulmonary disease (COPD), although their findings were not significant in the GLP-1 RA cohort. This behavior is undoubtedly influenced by patients with COPD having lower persistence rates compared to those with other chronic diseases [46,47]. The literature shows a negative correlation between treatment persistence and psychological disorders, such as anxiety and depression, in patients with T2DM [52,53,54]. Additionally, anxiety has been identified as a predictor of non-adherence [55,56], although in one study the association was observed only among males [57]. One study observed that patients with T2DM and obesity on GLP-1 RAs were more likely to discontinue therapy [49], while another study observed the opposite [58]. However, comparisons between studies may be affected by different definitions of discontinuation, with gaps between refills of ≥90 days [49] or ≥135 days [58]. No evidence of the influence of obesity was found in the SGLT-2i cohort.

For patients with established CVD, the predictors of non-persistence include newly diagnosed T2DM, insulin use, chronic respiratory disease, and anxiety. These findings are clinically relevant for several reasons. First, patients with newly diagnosed T2DM have a higher risk of macrovascular complications and mortality [51]. Second, the use of insulin in T2DM is associated with severe hyperglycemia, failed treatment with 2–3 OADs, or comorbidities such as CKD or liver cirrhosis, among others [59]. Fundamentally, these are factors that negatively impact the long-term prognosis for these patients [60].

Our study is subject to the limitations inherent in observational studies of this nature [61]. As in previous studies [44,47], patients with fewer than two prescription refills were excluded. Therefore, the study cohort does not represent all patients who initiated SGLT-2is or GLP-1 RAs, which may overestimate adherence and persistence rates. This potential bias should be considered when comparing our results to those of other studies. We considered drug switching within the same class. Therefore, the reported persistence should be interpreted as persistence with the index drug, not the medication class [44,47]. We have assumed that dispensing is equivalent to consumption, but this is not always true [62]. However, this is a widely accepted approach [28,31,32,44,47,63,64,65]. As in other studies [28,31,32,43], we have not obtained information about the underlying reasons for non-adherence and non-persistence. Specifically, no data are available on side effects, which may be a handicap, since genital infections in patients on SGLT-2is [66] and gastrointestinal symptoms in patients on oral semaglutide [39] may contribute to lower adherence and persistence. CONCYLIA did not record in-hospital medication use, which may have underestimated adherence and/or persistence for patients admitted during the 1-year follow-up period. Our database does not include clinical outcomes such as HbA1c levels, estimated glomerular filtration rate (eGFR), albumin–creatinine ratio (ACR), or body mass index (BMI). Consequently, the clinical utility of the observed differences in adherence and persistence rates between the SGLT-2i and GLP-1 RA cohorts has not been determined. SGLT-2is and GLP-1 RAs differ in dosing frequency and route of administration, which can directly affect treatment adherence and persistence [67]. Our dataset does not contain information on death data, which may affect the assessment of adherence and persistence. As shown in Table S1, the GLP-1 RA cohort included oral and injectable formulations with different dosing frequencies (daily and weekly). Furthermore, depending on the year of the index date, adherence and persistence in the GLP-1 RA cohort may have been influenced by the global shortage of semaglutide injectables [21,22]. Finally, although the robustness of our findings is supported using a 1:1 propensity score-matched design, residual biases cannot be excluded.

## 4. Materials and Methods

### 4.1. Study Design and Data Source

We conducted a retrospective, propensity score-matched cohort study in Castilla y León, a Spanish region with 2,401,221 inhabitants [68]. This study was designed according to the Strengthening the Reporting of Observational Studies in Epidemiology (STROBE) [69] and the Reporting of Studies Conducted using Observational Routinely collected health Data for Pharmacoepidemiology (RECORD-PE) [70] recommendations.

The data used in this study were obtained from the public health system claims database in Castilla y León (CONCYLIA), encompassing 97% of the population [71]. CONCYLIA contains health care information, including primary care prescribing and dispensing data, disease diagnosis codes based on the 10th revision of the International Classification of Diseases (ICD-10) [72], and sociodemographic data. The Pharmacy Directorate of the Castilla y León Health Council granted access to the data on 3 February 2023. Access to the data was granted pending ethical approval.

Since CONCYLIA provided de-identified data, patient informed consent was not required. The Ethics Committee of the Valladolid Health Areas approved this study on 27 July 2023 (code reference: PI-GR-3265 AP).

### 4.2. Cohort Selection

We selected patients with a diagnosis of T2DM who initiated treatment with SGLT-2is or GLP-1 RAs, either as monotherapy or in combination with other OADs, between 1 January 2019 and 31 December 2024. As in other studies [32], participants aged 40 or older were included to avoid misclassifying T2DM as another type of diabetes typically associated with younger people, such as T1DM, latent autoimmune diabetes of adults (LADA), and maturity-onset diabetes of the young (MODY) [73]. Supplementary Table S1 lists the SGLT-2is and GLP-1 RAs available in Spain during the study follow-up period (Anatomical Therapeutic Chemical [ATC] [74] subgroups A10BD, A10BJ and A10BK).

The index date was defined as the date of the first SGLT-2i or GLP-1 RA dispensation. Patients with less than two prescription refills of SGLT-2is or GLP-1 RAs, less than 12 months of follow-up, or inconsistent medication records were excluded. The final cohort of patients was followed for a 1-year period from the index date.

### 4.3. Outcomes

The primary outcomes were adherence and persistence on SGLT-2is and GLP-1 RAs. The Medication Possession Ratio (MPR) was used as a measure of adherence. MPR was calculated as the number of days’ supply during a specified follow-up period divided by the number of days from the first dispensing to switching, discontinuation or the end of the follow-up period, as first occurred [75]. The prescribed daily dose and quantity dispensed were used to estimate the duration of supply for each claim. For injectable medications, the potential waste generated in the dispensing container was given due consideration. In instances of overlapping claims, the early refill dates were adjusted to the day after the supply end date of the previous refill. Fixed-dose combinations were counted as a single drug. As in other studies [28,35], the calculation of MPR did not consider if the patient was admitted to the hospital during the follow-up period. In this case, it was assumed that the patient was covered by medication during the hospital stay. MPR values were not restricted to the maximum value of 100%, allowing figures to exceed this limit in patients with medication stockpiling. MPRs greater than 120% may be referred to as “over-adherence” and could have clinical relevance [76]. Finally, adherence levels were categorized according to MPR values as follows: none (<20), poor (20–49), moderate (50–79) and adherent (≥80) [65].

Persistence was defined as the time between the index date and treatment discontinuation. Discontinuation was defined as a gap ≥ 60 days between prescription refills of any SGLT-2i or GLP-1 RA. The discontinuation date was defined as the last day covered by the dispensation preceding the start of the ≥60-day gap without a refill. The ≥60-day gap was established in accordance with previous studies [44,47] and because this gap is used as a benchmark in our regional health system. The percentage of persistent patients at 3, 6, and 12 months was calculated. The total treatment persistence in days was calculated using the mean and its corresponding 95% confidence interval. The estimate of this measure is limited to the longest survival time, if it is censored. Drug switching (different ATC codes) within the same medication class (SGLT-2i or GLP-1 RA) was considered as discontinuation. Sensitivity analyses were conducted by changing the gap between prescription refills from 60 to 90 days.

### 4.4. Covariates

Covariates included sociodemographic data (sex, age, healthcare area and socioeconomic level), other treatments for T2DM (OADs and/or insulin), concomitant medication (polypharmacy), and chronic comorbidities. Patients with newly diagnosed T2DM were defined as those who had not received OADs (ATC subgroup A10B) 12 months before study entry. These covariates were used to balance the baseline characteristics between the SGLT-2i and GPL1-RA cohorts.

For the selection of patients with CVD, we focused on AF, IHD, HF, and ischemic stroke (IS) [77]. All diagnoses were based on ICD-10 codes (Supplementary Table S4).

### 4.5. Statistical Analyses

To ensure balanced cohorts, 1:1 propensity score-matched (PSM) was performed using a nearest neighbor matching algorithm with a 0.01 caliper. The propensity score was calculated using a logistic regression model that included sociodemographic, clinical, and comorbidity covariates. No missing data were recorded for these covariates. A total of 62 patients who did not have an adequate match were excluded from the matched cohort. SMD was calculated to compare the baseline characteristics of the SGLT-2i and GLP-1 RA cohorts. An SMD > 0.1 was considered to reveal significant differences between the two cohorts. The balance of covariates before and after matching is shown in Supplementary Figure S3.

Descriptive statistics were reported using percentages with their 95% confidence intervals (95% CIs) or means with their standard deviations (SDs), as appropriate. The sample’s normality was confirmed using Kolmogorov–Smirnov and Shapiro–Wilk tests. Differences between groups were analyzed using Student’s *t*-test and Pearson’s chi-squared test (χ^2^) for continuous and categorical variables, respectively.

Factors impacting adherence were analyzed using binary logistic regression. Treatment duration was assessed by Kaplan–Meier survival analysis and the log-rank test. The data were censored due to the absence of the event (discontinuation). Persistence was compared using Cox proportional hazards regression. HR values < 1 indicate a lower hazard of discontinuation and, therefore, a higher probability of persistence. Univariate and multivariate analyses were performed. All study covariates were included in the analyses. To verify the validity of the model, the Cox proportional hazards assumption was assessed using the Schoenfeld residual test. Additionally, the model’s goodness of fit was evaluated using the Cox–Snell residual plot. If the cumulative hazard function closely followed the 45° line, the final model was considered to exhibit an optimal fit.

SPSS version 29.0 (SPSS Inc., Chicago, IL, USA) and R version 2024.09.1 (R Project for Statistical Computing) were used for the statistical analyses. Statistical significance was set at *p* ≤ 0.05.

## 5. Conclusions

In conclusion, the SGLT-2i cohort showed higher adherence and persistence rates compared with the GLP-1 RA cohort. The difference in outcomes in favor of the SGLT-2i cohort was more pronounced among patients with CVD. This is clinically relevant since a 10% increase in adherence has a positive effect on cardiovascular outcomes and mortality [78,79]. Therefore, although the use of these drugs has been shown to reduce cardiovascular mortality and MACE in clinical trials [10,11,12,13], our evaluation focused exclusively on medication-taking behavior. In this context, the use of SGLT-2is proved to be more effective than GLP-1 RAs.

Understanding these findings is key to developing strategies that improve adherence, persistence, and ultimately, optimal glycemic control and reduced cardiovascular risk in patients with T2DM. According to the findings of our study, these strategies should primarily target patients with newly diagnosed T2DM who use insulin and have comorbidities such as chronic respiratory disease, anxiety, and obesity. To achieve this goal, incorporating adherence and persistence indicators into our regional public health system is particularly relevant. Finally, the use of these indicators has proven valuable for clinicians in previous studies [44,47,63,64,65].

## Figures and Tables

**Table 1:** Baseline characteristics.

	Original Cohort			PSM Cohort		
	**SGLT-2i**	**GLP-1 RA**	**SMD**	**SGLT-2i**	**GLP-1 RA**	**SMD**
**N**	45,102	7561		7499	7499	
**Sociodemographic characteristics**						
*Sex*						
Male	27,647 (61.3)	3860 (51.05)	0.208	3837 (51.17)	3847 (51.3)	0.003
*Age (mean ± SD)*	72.51 ± 11.47	65.80 ± 10.83	0.602	65.89 ± 10.89	65.91 ± 10.78	0.002
*Distribution by age groups*						
40–59	6196 (13.74)	2147 (28.4)	0.554	2091 (27.88)	2097 (27.96)	0.015
60–79	25,562 (56.68)	4614 (61.02)		4642 (61.9)	4603 (61.38)	
≥80	13,344 (29.59)	800 (10.58)		766 (10.21)	799 (10.65)	
Healthcare area						
Urban	23,446 (51.98)	4100 (54.23)	0.045	4122 (54.97)	4067 (54.23)	0.015
*Socioeconomic level* (€/year)						
<18,000	29,782 (66.03)	4787 (63.31)	0.071	4703 (62.72)	4749 (63.33)	0.019
18,000–100,000	14,988 (33.23)	2681 (35.46)		2715 (36.2)	2659 (35.46)	
>100,000	332 (0.74)	93 (1.23)		81 (1.08)	91 (1.21)	
**Clinical characteristics**						
Polypharmacy (≥5 drugs)	42,685 (94.64)	7331 (96.96)	0.116	7263 (96.85)	7269 (96.93)	0.005
Newly diagnosed T2DM	14,571 (32.31)	2076 (27.46)	0.106	2018 (26.91)	2056 (27.42)	0.011
Monotherapy	5507 (12.21)	432 (5.71)	0.229	383 (5.11)	431 (5.75)	0.028
*Other Antidiabetic agents*						
Metformin	25,213 (59.34)	4986 (71.19)	0.207	4972 (67.92)	4933 (71.03)	0.011
DPP-4i	14,508 (34.14)	1691 (24.14)	0.221	1737 (23.73)	1688 (24.31)	0.016
Sulphonylureas	5998 (14.12)	807 (11.52)	0.081	851 (11.63)	805 (11.59)	0.02
Glinide	4275 (10.06)	718 (10.25)	0.001	702 (9.59)	710 (10.22)	0.004
Glitazones	513 (1.21)	236 (3.37)	0.138	201 (2.75)	226 (3.25)	0.02
Acarbose	189 (0.44)	10 (0.14)	0.055	9 (0.12)	10 (0.14)	0.004
Insulin use	12,287 (27.24)	3073 (40.64)	0.286	3065 (40.87)	3037 (40.5)	0.008
Switching	5740 (12.73)	490 (6.48)	0.213	404 (5.39)	489 (6.52)	0.048
*Other drugs*						
Anti-lipemic agents	35,096 (83.05)	5925 (84.76)	0.013	5843 (84.12)	5883 (84.81)	0.013
Angiotensin II inhibitors	18,033 (42.67)	3307 (47.31)	0.076	3187 (45.88)	3272 (47.17)	0.023
Platelet aggregation inhibitors	18,373 (43.48)	2540 (36.34)	0.148	2480 (35.7)	2528 (36.44)	0.014
β blockers	13,671 (32.35)	1777 (25.42)	0.154	1717 (24.72)	1769 (25.5)	0.016
ACEi	12,425 (29.4)	1948 (27.87)	0.04	1940 (27.93)	1935 (27.89)	0.002
Loop diuretics	10,659 (25.22)	1513 (21.65)	0.088	1372 (19.75)	1494 (21.54)	0.041
Calcium channel blockers	7747 (18.33)	1317 (18.84)	0.006	1337 (19.25)	1309 (18.87)	0.01
Potassium sparing diuretics	4537 (10.74)	416 (5.95)	0.171	383 (5.51)	415 (5.98)	0.019
Nitrates	2387 (5.65)	305 (4.36)	0.06	258 (3.71)	305 (4.4)	0.033
**Comorbidities**						
CVD	15,159 (33.61)	1671 (22.1)	0.259	1634 (21.79)	1668 (22.24)	0.011
Atrial fibrillation	7701 (18.47)	703 (10)	0.231	693 (9.93)	701 (10.06)	0.004
Ischemic Heart Disease	5909 (14.17)	773 (10.99)	0.09	754 (10.8)	773 (11.09)	0.008
Heart failure	5751 (13.79)	437 (6.22)	0.242	390 (5.59)	435 (6.24)	0.026
Ischemic Stroke	1659 (3.98)	191 (2.72)	0.066	197 (2.82)	191 (2.74)	0.005
Hypertension	27,311 (65.51)	4877 (69.36)	0.082	4838 (69.31)	4833 (69.33)	0.001
Hyperlipidemia	25,555 (61.3)	4602 (65.45)	0.085	4581 (65.63)	4567 (65.51)	0.004
Chronic respiratory disease	10,925 (26.21)	1971 (28.03)	0.043	1883 (26.98)	1945 (27.9)	0.019
Anxiety	6681 (16.03)	1400 (19.91)	0.099	1343 (19.24)	1375 (19.72)	0.011
Depression	3884 (9.32)	765 (10.88)	0.052	754 (10.8)	749 (10.74)	0.002
Chronic Kidney Disease	1643 (3.94)	271 (3.85)	0.003	247 (3.54)	269 (3.86)	0.016
Gout	1307 (3.14)	174 (2.47)	0.037	165 (2.36)	174 (2.5)	0.008
Obesity	344 (0.83)	311 (4.42)	0.219	196 (2.81)	273 (3.92)	0.059

Abbreviations: PSM, propensity score-matched, SGLT-2is, sodium-glucose co-transporter 2 inhibitors, GLP-1 RAs, glucagon-like peptide-1 analogues, DPP-4is, dipeptidyl peptidase 4 inhibitors, SMD, standardized mean difference, SD, standard deviation, ACEis, angiotensin converting enzyme inhibitors, CVD, cardiovascular disease.

**Table 2:** Adherence and persistence during 1-year follow-up.

	PSM Cohort		
	SGLT-2i	GLP-1 RA	*p*
N	7499	7499	
Adherence % (95% CI)			
MPR (mean ± SD)	91.13 ± 13.39	87.13 ± 20.86	0.001
Adherence prevalence	86.66 (85.89–87.43)	76.53 (75.57–77.49)	0.001
Adherence level			
None (<20)	0.25 (0.14–0.36)	0.21 (0.11–0.31)	0.001
Poor (20–49)	2.45 (2.1–2.8)	1.91 (1.6–2.22)	
Moderate (50–79)	10.63 (9.93–11.33)	21.35 (20.42–22.28)	
OR for adherence * (95% CI)	2.02 (1.86–2.21)	1 (Reference)	0.001
Persistence % (95% CI)			
3 months	89.96 (89.28–90.64)	87.93 (87.19–88.67)	0.001
6 months	82.28 (81.42–83.14)	79.31 (78.39–80.23)	0.001
12 months	71.48 (70.46–72.5)	66.52 (65.45–67.59)	0.001
Treatment persistence (days) (mean (95% CI)	303.76 (301.28–306.25)	294.22 (291.58–296.85)	0.001
HR for discontinuation ** (95% CI)	0.83 (0.78–0.88)	1 (Reference)	0.001

* Logistic regression model (multivariate analysis). ** Cox proportional hazards model (multivariate analysis). Abbreviations: PSM, propensity score-matched; SGLT-2is, sodium-glucose co-transporter 2 inhibitors; GLP-1 RAs, glucagon-like peptide-1 analogues; CI, confidence interval; SD, standard deviation; OR, odds ratio; HR, hazard ratio.

**Table 3:** Cox proportional hazards regression (after PSM) for discontinuation in patients with T2DM on sodium-glucose cotransporter-2 inhibitors (SGLT-2is) and glucagon-like peptide-1 receptor agonists (GLP-1 RAs).

	Univariate Analysis		Multivariate Analysis	
Variables	HR (95% CI)	*p*	HR (95% CI)	*p*
Cohort (SGLT-2i)	0.79 (0.76–0.86)	0.001	0.83 (0.78–0.88)	0.001
**Sociodemographic characteristics**				
Sex (male)	0.87 (0.83–0.93)	0.001	0.9 (0.85–0.96)	0.001
Age	1.01 (1.01–1-01)	0.045	1 (1–1.01)	0.057
Healthcare area (Urban)	1.04 (0.98–1.10)	0,21	1.06 (1–1.12)	0.053
Socioeconomical level (€/year)				
<18,000	1 (Reference)		1 (Reference)	
18,000–100,000	0.59 (0.43–0.82)	0.002	0.65 (0.47–0.9)	0.001
>100,000	0.72 (0.52–1.01)	0.057	0.76 (0.55–1.06)	0.115
**Clinical characteristics**				
Newly diagnosed T2DM (Yes)	1.18 (1.11–1.25)	0.001	1.25 (1.16–1.33)	0.001
Polypharmacy (Yes)	1.1 (0.93–1.3)	0.278	1.09 (0.91–1.3)	0.379
Monotherapy (Yes)	0.89 (0.78–1.02)	0.094	0.81 (0.7–0.92)	0.002
Insulin use (Yes)	1.23 (1.16–1.3)	0.001	1.32 (1.23–1.39)	0.001
**Comorbidities**				
CVD (Yes)	1.04 (0.97–1.11)	0.283	1.06 (0.98–1.14)	0.128
Hypertension (Yes)	0.93 (0.88–0.99)	0.028	0.96 (0.91–1.02)	0.248
Hyperlipidemia (Yes)	0.88 (0.83–0.93)	0.001	0.89 (0.84–0.94)	0.001
Chronic airways disease (Yes)	1.11 (1.04–1.19)	0.001	1.08 (1.01–1.15)	0.023
Anxiety (Yes)	1.19 (1.10–1.27)	0.001	1.12 (1.04–1.20)	0.002
Depression (Yes)	1.05 (0.95–1.15)	0.296	1.00 (0.91–1.10)	0.995
Chronic Kidney Disease (Yes)	1.16 (1.01–1.35)	0.044	1.15 (0.99–1.35)	0.061
Gout (Yes)	0.94 (0.78–1.15)	0.584	1.00 (0.82–1.22)	0.978
Obesity (Yes)	1.25 (1.08–1.45)	0.004	1.18 (1.01–1.37)	0.037

Abbreviations: SGLT-2is, sodium-glucose co-transporter 2 inhibitors, CI, confidence interval, HR, hazard ratio, CVD, cardiovascular disease.

**Table 4:** Adherence and persistence during 1-year follow-up in patients with T2DM and cardiovascular disease.

Subgroup ^a^	N per Cohort	Adherence Prevalence (%) SGLT-2i vs. GLP-1 RA	OR * (95% CI)	*p*	Persistence Prevalence (%) SGLT-2i vs. GLP-1 RA	HR ** (95% CI)	*p*
Overall CVD patients	1659	89.57 vs. 78.12	2.44 (2.01–2.97)	0.001	73.9 vs. 63.59	0.66 (0.58–0.75)	0.001
Atrial fibrillation	693	90.91 vs. 79.8	2.64 (1.91–3.65)	0.001	74.17 vs. 66.52	0.74 (0.61–0.90)	0.003
Ischemic heart disease	773	89 vs. 77.75	2.34 (1.76–3.10)	0.001	72.7 vs. 63	0.69 (0.58–0.83)	0.001
Heart failure	427	87.12 vs. 74.94	2.28 (1.58–3.27)	0.001	68.85 vs. 60.89	0.75 (0.60–0.94)	0.014
Ischemic stroke	188	88.3 vs. 80.32	1.91 (1.07–3.39)	0.029	72.87 vs. 57.45	0.59 (0.41–0.83)	0.003

^a^ Separate propensity-matched cohorts were generated for each subgroup analysis. * Logistic regression model (multivariate analysis). Reference (OR = 1; GLP-1 RA). ** Cox proportional hazards model (multivariate analysis). HR for discontinuation. Reference (HR = 1; GLP-1 RA). Abbreviations: CVD, cardiovascular disease, SGLT2is, sodium-glucose co-transporter 2 inhibitors, GLP-1 RAs, glucagon-like peptide-1 analogues, CI, confidence interval, SD, standard deviation, OR, odds ratio, HR, hazard ratio.

## Data Availability

The data presented in this study are available on request from the corresponding author due to ethical restrictions.

## Supplementary Materials

The following supporting information can be downloaded at https://www.mdpi.com/article/10.3390/ph19101607/s1, Figure S1: Cox–Snell residual plots on patients with T2DM on sodium-glucose cotransporter-2 inhibitors (SGLT-2is) and glucagon-like peptide-1 receptor agonists (GLP-1 RAs). (a) All patients, (b) patients with cardiovascular disease; Figure S2: Kaplan–Meier analysis for treatment persistence by type of cardiovascular disease (CVD) in patients on sodium-glucose cotransporter-2 inhibitors (SGLT-2is) and glucagon-like peptide-1 receptor agonists (GLP-1 RAs); Figure S3: Standardized mean differences in baseline covariates before and after propensity score matching (Love Plot); Table S1: SGLT-2is and GLP-1 RAs available in Spain and patients on treatment during the follow-up period; Table S2: Adherence and persistence during 1-year follow-up (original cohort); Table S3: Cox proportional hazards regression (after PSM) for discontinuation in patients with T2DM and cardiovascular disease (CVD) on sodium-glucose cotransporter-2 inhibitors (SGLT-2is) and glucagon-like peptide-1 receptor agonists (GLP-1 RAs); Table S4: International Classification of Disease 10th edition (ICD-10) codes used to define the comorbidities in the study cohort.

## References

[1] Khan M.A.B., Hashim M.J., King J.K., Govender R.D., Mustafa H., Al Kaabi J. (2020). Epidemiology of Type 2 Diabetes—Global Burden of Disease and Forecasted Trends. J. Epidemiol. Glob. Health.

[2] International Diabetes Federation IDF Diabetes Atlas. https://diabetesatlas.org/resources/idf-diabetes-atlas-2025/.

[3] Essien U.R., Singh B., Swabe G., Johnson A.E., Eberly L.A., Wadhera R.K., Breathett K., Vaduganathan M., Magnani J.W. (2023). Association of Prescription Co-Payment with Adherence to Glucagon-like Peptide-1 Receptor Agonist and Sodium-Glucose Cotransporter-2 Inhibitor Therapies in Patients with Heart Failure and Diabetes. JAMA Netw. Open.

[4] Fonseca V.A. (2009). Defining and Characterizing the Progression of Type 2 Diabetes. Diabetes Care.

[5] Dunlay S.M., Givertz M.M., Aguilar D., Allen L.A., Chan M., Desai A.S., Deswal A., Dickson V.V., Kosiborod M.N., Lekavich C.L. (2019). Type 2 Diabetes Mellitus and Heart Failure: A Scientific Statement from the American Heart Association and the Heart Failure Society of America: This Statement Does Not Represent an Update of the 2017 ACC/AHA/HFSA Heart Failure Guideline Update. Circulation.

[6] Ormazabal V., Nair S., Elfeky O., Aguayo C., Salomon C., Zuñiga F.A. (2018). Association between Insulin Resistance and the Development of Cardiovascular Disease. Cardiovasc. Diabetol..

[7] Fox C.S. (2010). Cardiovascular Disease Risk Factors, Type 2 Diabetes Mellitus, and the Framingham Heart Study. Trends Cardiovasc. Med..

[8] Almdal T., Scharling H., Jensen J.S., Vestergaard H. (2004). The Independent Effect of Type 2 Diabetes Mellitus on Ischemic Heart Disease, Stroke, and Death: A Population-Based Study of 13,000 Men and Women with 20 Years of Follow-Up. Arch. Intern. Med..

[9] Kirchhof P., Benussi S., Kotecha D., Ahlsson A., Atar D., Casadei B., Castella M., Diener H.-C., Heidbuchel H., Hendriks J. (2016). 2016 ESC Guidelines for the Management of Atrial Fibrillation Developed in Collaboration with EACTS. Eur. Heart J..

[10] Zinman B., Wanner C., Lachin J.M., Fitchett D., Bluhmki E., Hantel S., Mattheus M., Devins T., Johansen O.E., Woerle H.J. (2015). Empagliflozin, Cardiovascular Outcomes, and Mortality in Type 2 Diabetes. N. Engl. J. Med..

[11] Neal B., Perkovic V., Mahaffey K.W., de Zeeuw D., Fulcher G., Erondu N., Shaw W., Law G., Desai M., Matthews D.R. (2017). Canagliflozin and Cardiovascular and Renal Events in Type 2 Diabetes. N. Engl. J. Med..

[12] Zelniker T.A., Wiviott S.D., Raz I., Im K., Goodrich E.L., Furtado R.H.M., Bonaca M.P., Mosenzon O., Kato E.T., Cahn A. (2019). Comparison of the Effects of Glucagon-Like Peptide Receptor Agonists and Sodium-Glucose Cotransporter 2 Inhibitors for Prevention of Major Adverse Cardiovascular and Renal Outcomes in Type 2 Diabetes Mellitus. Circulation.

[13] Newman J.D., Vani A.K., Aleman J.O., Weintraub H.S., Berger J.S., Schwartzbard A.Z. (2018). The Changing Landscape of Diabetes Therapy for Cardiovascular Risk Reduction: JACC State-of-the-Art Review. J. Am. Coll. Cardiol..

[14] Davies M.J., Aroda V.R., Collins B.S., Gabbay R.A., Green J., Maruthur N.M., Rosas S.E., Del Prato S., Mathieu C., Mingrone G. (2022). Management of Hyperglycaemia in Type 2 Diabetes, 2022. A Consensus Report by the American Diabetes Association (ADA) and the European Association for the Study of Diabetes (EASD). Diabetologia.

[15] Ho P.M., Rumsfeld J.S., Masoudi F.A., McClure D.L., Plomondon M.E., Steiner J.F., Magid D.J. (2006). Effect of Medication Nonadherence on Hospitalization and Mortality among Patients with Diabetes Mellitus. Arch. Intern. Med..

[16] Kim Y.-Y., Lee J.-S., Kang H.-J., Park S.M. (2018). Effect of Medication Adherence on Long-Term All-Cause-Mortality and Hospitalization for Cardiovascular Disease in 65,067 Newly Diagnosed Type 2 Diabetes Patients. Sci. Rep..

[17] Skriver L.K.L., Nielsen M.W., Walther S., Nørlev J.D., Hangaard S. (2023). Factors Associated with Adherence or Nonadherence to Insulin Therapy among Adults with Type 2 Diabetes Mellitus: A Scoping Review. J. Diabetes Complicat..

[18] Polonsky W.H., Henry R.R. (2016). Poor Medication Adherence in Type 2 Diabetes: Recognizing the Scope of the Problem and Its Key Contributors. Patient Prefer. Adherence.

[19] Johnson C.E., Sussman W.B., Weeda E.R. (2024). Medication Adherence to Sodium-Glucose Cotransporter-2 Inhibitors versus Glucagon-like Peptide-1 Receptor Agonists: A Meta-Analysis. Diabetes Obes. Metab..

[20] Ciardullo S., Savaré L., Rea F., Perseghin G., Corrao G. (2024). Adherence to GLP1-RA and SGLT2-I Affects Clinical Outcomes and Costs in Patients with Type 2 Diabetes. Diabetes Metab. Res. Rev..

[21] Spanish Agency for Medicines and Health Products (AEMPS) Supply of GLP-1 Analogues. https://www.aemps.gob.es/informa/la-aemps-actualiza-la-situacion-de-suministro-de-los-medicamentos-analogos-del-glp-1-2/.

[22] U.S. Food & Drug Administration (FDA) FDA Clarifies Policies for Compounders as National GLP-1 Supply Begins to Stabilize. https://www.fda.gov/drugs/drug-alerts-and-statements/fda-clarifies-policies-compounders-national-glp-1-supply-begins-stabilize.

[23] European Medicines Agency (EMA) Rybelsus: EPAR—Medicine Overview (EMA/62316/2020). https://www.ema.europa.eu/en/documents/overview/rybelsus-epar-medicine-overview_en.pdf.

[24] Arnold S.V., Gosch K., Kosiborod M., Wong N.D., Sperling L.S., Newman J.D., Gamble C.L., Hamersky C., Rajpura J., Vaduganathan M. (2023). Contemporary Use of Cardiovascular Risk Reduction Strategies in Type 2 Diabetes. Insights from the Diabetes Collaborative Registry. Am. Heart J..

[25] King A., Tan X., Dhopeshwarkar N., Bohn R., Dea K., Leonard C.E., de Havenon A. (2024). Recent Trends in GLP-1 RA and SGLT2i Use among People with Type 2 Diabetes and Atherosclerotic Cardiovascular Disease in the USA. BMJ Open Diabetes Res. Care.

[26] Jermendy G., Kiss Z., Rokszin G., Abonyi-Tóth Z., Wittmann I., Kempler P. (2018). Persistence to Treatment with Novel Antidiabetic Drugs (Dipeptidyl Peptidase-4 Inhibitors, Sodium-Glucose Co-Transporter-2 Inhibitors, and Glucagon-like Peptide-1 Receptor Agonists) in People with Type 2 Diabetes: A Nationwide Cohort Study. Diabetes Ther. Res. Treat. Educ. Diabetes Relat. Disord..

[27] O’Hara D.V., Janse R.J., Fu E.L., Jardine M.J., Carrero J.-J. (2024). Adherence and Persistence to Novel Glucose-Lowering Medications in Persons with Type 2 Diabetes Mellitus Undergoing Routine Care. Diabetes Res. Clin. Pract..

[28] Malik M.E., Falkentoft A.C., Jensen J., Zahir D., Parveen S., Alhakak A., Andersson C., Petrie M.C., Sattar N., McMurray J.J.V. (2023). Discontinuation and Reinitiation of SGLT-2 Inhibitors and GLP-1R Agonists in Patients with Type 2 Diabetes: A Nationwide Study from 2013 to 2021. Lancet Reg. Health Eur..

[29] Gelzer A.D., Gao W., Keleti D., Donia T., Megargell L., Kreitman J., Michael K.E. (2019). Multifaceted Interventions Improve Medication Adherence and Reduce Acute Hospitalization Rates in Medicaid Patients Prescribed Asthma Controllers. J. Asthma Off. J. Assoc. Care Asthma.

[30] Cai J., Divino V., Burudpakdee C. (2017). Adherence and Persistence in Patients with Type 2 Diabetes Mellitus Newly Initiating Canagliflozin, Dapagliflozin, Dpp-4s, or Glp-1s in the United States. Curr. Med. Res. Opin..

[31] Rea F., Ciardullo S., Savaré L., Perseghin G., Corrao G. (2021). Comparing Medication Persistence among Patients with Type 2 Diabetes Using Sodium-Glucose Cotransporter 2 Inhibitors or Glucagon-like Peptide-1 Receptor Agonists in Real-World Setting. Diabetes Res. Clin. Pract..

[32] Donnelly L.A., Singh K., McCrimmon R.J., Pearson E.R. (2026). One-Year Usage Patterns of SGLT-2 Inhibitors and GLP-1 Receptor Agonists in Individuals with Type 2 Diabetes in a Real-World Population. Diabetes Obes. Metab..

[33] Ofori-Asenso R., Sahle B.W., Chin K.L., Mazidi M., Ademi Z., De Bruin M.L., Liew D. (2021). Poor Adherence and Persistence to Sodium Glucose Co-Transporter 2 Inhibitors in Real-World Settings: Evidence from a Systematic Review and Meta-Analysis. Diabetes Metab. Res. Rev..

[34] Svensson A.-M., Toll A., Lebrec J., Miftaraj M., Franzén S., Eliasson B. (2021). Treatment Persistence in Patients with Type 2 Diabetes Treated with Glucagon-like Peptide-1 Receptor Agonists in Clinical Practice in Sweden. Diabetes Obes. Metab..

[35] Lassen M.C.H., Johansen N.D., Modin D., Catarig A.-M., Vistisen B.K., Amadid H., Zimmermann E., Gislason G., Biering-Sørensen T. (2024). Adherence to Glucagon-like Peptide-1 Receptor Agonist Treatment in Type 2 Diabetes Mellitus: A Nationwide Registry Study. Diabetes Obes. Metab..

[36] Kassem S., Khalaila B., Stein N., Saliba W., Zaina A. (2024). Efficacy, Adherence and Persistence of Various Glucagon-like Peptide-1 Agonists: Nationwide Real-Life Data. Diabetes Obes. Metab..

[37] Almandoz J.P., Lingvay I., Morales J., Campos C. (2020). Switching Between Glucagon-Like Peptide-1 Receptor Agonists: Rationale and Practical Guidance. Clin. Diabetes Publ. Am. Diabetes Assoc..

[38] European Medicines Agency (EMA) Rybelsus: EPAR—Product Information. https://www.ema.europa.eu/en/documents/product-information/rybelsus-epar-product-information_en.pdf.

[39] Conti M., Pontiggia L., Vergani M., Muraca E., Cannistraci R., Perra S., Lattuada G., Perseghin G., Ciardullo S. (2025). Comparing Medication Persistence with Oral and Subcutaneous Semaglutide in a Real-World Setting. Acta Diabetol..

[40] World Health Organization (WHO) Adherence to Long-Term Therapies: Evidence for Action. https://www.paho.org/en/documents/who-adherence-long-term-therapies-evidence-action-2003.

[41] Boye K., Ross M., Mody R., Konig M., Gelhorn H. (2021). Patients’ Preferences for Once-Daily Oral versus Once-Weekly Injectable Diabetes Medications: The REVISE Study. Diabetes Obes. Metab..

[42] Qin L., Chen S., Flood E., Shaunik A., Romero B., de la Cruz M., Alvarez C., Grandy S. (2017). Glucagon-like Peptide-1 Receptor Agonist Treatment Attributes Important to Injection-Experienced Patients with Type 2 Diabetes Mellitus: A Preference Study in Germany and the United Kingdom. Diabetes Ther. Res. Treat. Educ. Diabetes Relat. Disord..

[43] Ofori-Asenso R., Liew D., Lalic S., Mazidi M., Magliano D.J., Ademi Z., Bell J.S., Ilomaki J. (2019). Adherence, Persistence, and Switching Among People Prescribed Sodium Glucose Co-Transporter 2 Inhibitors: A Nationwide Retrospective Cohort Study. Adv. Ther..

[44] Martín-Fernández M., González-González M.A., Pedrosa-Naudín M.A., Fernández-Lázaro D., Álvarez F.J., Gutiérrez-Abejón E. (2025). Newly Started Versus Previously Treated Statin Patients: A Retrospective Cohort Study Comparing Adherence and Persistence with Reference to Cardiovascular Prevention. Pharmaceuticals.

[45] American Diabetes Association (2020). Pharmacologic Approaches to Glycemic Treatment: Standards of Medical Care in Diabetes-2020. Diabetes Care.

[46] Rolnick S.J., Pawloski P.A., Hedblom B.D., Asche S.E., Bruzek R.J. (2013). Patient Characteristics Associated with Medication Adherence. Clin. Med. Res..

[47] González-González M.A., Pedrosa-Naudín M.A., Fernández-Lázaro D., Planelles I.D., Álvarez F.J., Gutiérrez-Abejón E. (2025). Single-versus Multiple-Inhaler Triple Therapy in Patients with COPD in Spain: A Retrospective Cohort Study Comparing Adherence, Persistence, Risk of Exacerbations and Economic Outcomes. Front. Pharmacol..

[48] Sikirica M.V., Martin A.A., Wood R., Leith A., Piercy J., Higgins V. (2017). Reasons for Discontinuation of GLP1 Receptor Agonists: Data from a Real-World Cross-Sectional Survey of Physicians and Their Patients with Type 2 Diabetes. Diabetes Metab. Syndr. Obes. Targets Ther..

[49] Weiss T., Carr R.D., Pal S., Yang L., Sawhney B., Boggs R., Rajpathak S., Iglay K. (2020). Real-World Adherence and Discontinuation of Glucagon-like Peptide-1 Receptor Agonists Therapy in Type 2 Diabetes Mellitus Patients in the United States. Patient Prefer. Adherence.

[50] Boonpattharatthiti K., Songkla P.N., Chantara J., Koomsri C., Krass I., Chaiyakunapruk N., Dhippayom T. (2024). Prevalence of Adherence to Oral Antidiabetic Drugs in Patients with Type 2 Diabetes: A Systematic Review and Meta-Analysis. J. Diabetes Investig..

[51] Kim N.H., Moon J.S., Ha K.H., Kim J., Kwon K.-E., Kim S.G., Kim D.J. (2025). First-Year Oral Antidiabetic Adherence and Long-Term Complications in Newly Diagnosed Type 2 Diabetes. Diabetes Res. Clin. Pract..

[52] Ahmed S., Saif-Ur-Rahman K.M., Dhungana R.R., Ganbaatar G., Ashraf F., Yano Y., Miura K., Ahmed M.S.A.M. (2023). Medication Adherence and Health-Related Quality of Life among People with Diabetes in Bangladesh: A Cross-Sectional Study. Endocrinol. Diabetes Metab..

[53] Ali S.A., Salih S.M., Abdelmola A., Makeen A.M., Solan Y.M., Mohammed Ali M.M. (2024). Association of Anxiety and Depressive Symptoms and Treatment Adherence Among Patients with Type 2 Diabetes Mellitus in Jazan, Saudi Arabia: A Cross-Sectional Study. Cureus.

[54] Świątoniowska-Lonc N., Tański W., Polański J., Jankowska-Polańska B., Mazur G. (2021). Psychosocial Determinants of Treatment Adherence in Patients with Type 2 Diabetes—A Review. Diabetes Metab. Syndr. Obes. Targets Ther..

[55] Mendes R., Martins S., Fernandes L. (2019). Adherence to Medication, Physical Activity and Diet in Older Adults with Diabetes: Its Association with Cognition, Anxiety and Depression. J. Clin. Med. Res..

[56] Krass I., Schieback P., Dhippayom T. (2015). Adherence to Diabetes Medication: A Systematic Review. Diabet. Med. J. Br. Diabet. Assoc..

[57] Lara-Morales A., Gandarillas-Grande A., Díaz-Holgado A., Serrano-Gallardo P. (2022). Psychosocial Factors in Adherence to Pharmacological Treatment and Diabetes Mellitus Control in Patients over 65. Aten. Primaria.

[58] Do D., Lee T., Peasah S.K., Good C.B., Inneh A., Patel U. (2024). GLP-1 Receptor Agonist Discontinuation Among Patients with Obesity and/or Type 2 Diabetes. JAMA Netw. Open.

[59] Galdón Sanz-Pastor A., Justel Enríquez A., Sánchez Bao A., Ampudia-Blasco F.J. (2024). Current Barriers to Initiating Insulin Therapy in Individuals with Type 2 Diabetes. Front. Endocrinol..

[60] Nielsen L.B., Bartholdy A., Nguyen T.-L., Jensen M.K., Rasmussen S., Guasch-Ferré M., Merino J. (2026). Long-Term Adherence to Glucose-Lowering Therapy and Cardiovascular Risk in Type 2 Diabetes: A Nationwide Cohort Study. Lancet Reg. Health Eur..

[61] Suissa S., Garbe E. (2007). Primer: Administrative Health Databases in Observational Studies of Drug Effects—Advantages and Disadvantages. Nat. Rev. Rheumatol..

[62] Corrao G., Mancia G. (2015). Generating Evidence from Computerized Healthcare Utilization Databases. Hypertension.

[63] Gutiérrez-Abejón E., Pedrosa-Naudín M.A., Fernández-Lázaro D., Alvarez F.J. (2023). Medication Economic Burden of Antidepressant Non-Adherence in Spain. Front. Pharmacol..

[64] Gutiérrez-Abejón E., Pedrosa-Naudín M.A., Fernández-Lázaro D., Díaz Planelles I., Álvarez F.J. (2024). Non-Adherence to Antidementia Medications and Associated Factors: A Study of Spanish Population-Based Registry Data. Front. Pharmacol..

[65] Pedrosa-Naudín M.A., Gutiérrez-Abejón E., Herrera-Gómez F., Fernández-Lázaro D., Álvarez F.J. (2022). Non-Adherence to Antidepressant Treatment and Related Factors in a Region of Spain: A Population-Based Registry Study. Pharmaceutics.

[66] Scheen A.J. (2019). An Update on the Safety of SGLT2 Inhibitors. Expert Opin. Drug Saf..

[67] Weeda E.R., Muraoka A.K., Brock M.D., Cannon J.M. (2021). Medication Adherence to Injectable Glucagon-like Peptide-1 (GLP-1) Receptor Agonists Dosed Once Weekly vs Once Daily in Patients with Type 2 Diabetes: A Meta-Analysis. Int. J. Clin. Pract..

[68] Spanish National Statistics Institute (INE) Demographics and Population. https://www.ine.es/dyngs/INEbase/categoria.htm?c=Estadistica_P&cid=1254734710990.

[69] von Elm E., Altman D.G., Egger M., Pocock S.J., Gøtzsche P.C., Vandenbroucke J.P., STROBE Initiative (2008). The Strengthening the Reporting of Observational Studies in Epidemiology (STROBE) Statement: Guidelines for Reporting Observational Studies. J. Clin. Epidemiol..

[70] Langan S.M., Schmidt S.A., Wing K., Ehrenstein V., Nicholls S.G., Filion K.B., Klungel O., Petersen I., Sorensen H.T., Dixon W.G. (2018). The Reporting of Studies Conducted Using Observational Routinely Collected Health Data Statement for Pharmacoepidemiology (RECORD-PE). BMJ.

[71] Gerencia Regional de Salud de Castilla y León CONCYLIA, Sistema de Información Para la Gestión de la Prestación Farmacéutica en SACYL. https://www.saludcastillayleon.es/portalmedicamento/es/indicadores-informes/concylia.

[72] World Health Organization (WHO) International Statistical Classification of Diseases and Related Health Problems, ICD-10 Volume 2. https://www.who.int/publications/m/item/international-statistical-classification-of-diseases-and-related-health-problems---volume-2.

[73] Strati M., Moustaki M., Psaltopoulou T., Vryonidou A., Paschou S.A. (2024). Early Onset Type 2 Diabetes Mellitus: An Update. Endocrine.

[74] World Health Organization (WHO) Collaborating Centre for Drug Statistics Methodology—ATC/DDD Index. https://www.whocc.no/atc_ddd_index/.

[75] Andrade S.E., Kahler K.H., Frech F., Chan K.A. (2006). Methods for Evaluation of Medication Adherence and Persistence Using Automated Databases. Pharmacoepidemiol. Drug Saf..

[76] Raebel M.A., Schmittdiel J., Karter A.J., Konieczny J.L., Steiner J.F. (2013). Standardizing Terminology and Definitions of Medication Adherence and Persistence in Research Employing Electronic Databases. Med. Care.

[77] Grundy S.M., Stone N.J., Bailey A.L., Beam C., Birtcher K.K., Blumenthal R.S., Braun L.T., de Ferranti S., Faiella-Tommasino J., Forman D.E. (2019). 2018 AHA/ACC/AACVPR/AAPA/ABC/ACPM/ADA/AGS/APhA/ASPC/NLA/PCNA Guideline on the Management of Blood Cholesterol: A Report of the American College of Cardiology/American Heart Association Task Force on Clinical Practice Guidelines. Circulation.

[78] Khunti K., Danese M.D., Kutikova L., Catterick D., Sorio-Vilela F., Gleeson M., Kondapally Seshasai S.R., Brownrigg J., Ray K.K. (2018). Association of a Combined Measure of Adherence and Treatment Intensity with Cardiovascular Outcomes in Patients with Atherosclerosis or Other Cardiovascular Risk Factors Treated with Statins and/or Ezetimibe. JAMA Netw. Open.

[79] Qin X., Hung J., Knuiman M.W., Briffa T.G., Teng T.-H.K., Sanfilippo F.M. (2023). Evidence-Based Medication Adherence among Seniors in the First Year after Heart Failure Hospitalisation and Subsequent Long-Term Outcomes: A Restricted Cubic Spline Analysis of Adherence-Outcome Relationships. Eur. J. Clin. Pharmacol..

