# Supplementary material for: SGLT-2 Inhibitors Versus GLP-1 Receptor Agonists in Patients with Type 2 Diabetes Mellitus: A Propensity Score-Matched Cohort Study Comparing Adherence and Persistence with Specific Reference to Cardiovascular Disease
Source: Pharmaceuticals (Basel). 2026 Oct 10;19(10):1607. doi: 10.3390/ph19101607 (PMC13708738; doi:10.3390/ph19101607)
Supplement: Supplementary file 1 [file pharmaceuticals-19-01607-s001.zip › pharmaceuticals-4565921-supplementary.pdf]

Table S1: Sodium-glucose cotransporter-2 inhibitors (SGLT-2is) and glucagon-like peptide-1 receptor agonists (GLP-1 RAs) available in Spain and patients on treatment during the follow-up period.

OAD type	ATC Code [74]	Name	Administration		Patients on treatment (N)[a]
			Route	Frequency	
GLP-1 RA	A10BJ06	Semaglutide	O	D	3265
			P	W	2465
	A10BJ05	Dulaglutide	P	W	1823
	A10BJ02	Liraglutide	P	D	351
	A10BJ01	Exenatide	P	W*	55
			P	D	6
	A10BJ03	Lixisenatide	P	D	23
SGLT-2i	A10BK01, A10BD15	Dapagliflozin, metformin and dapagliflozin.	O	D	3655
	A10BK03, A10BD19, A10BD20	Empagliflozin, linagliptin and empagliflozin, metformin and empagliflozin			3636
	A10BK02, A10BD16	Canagliflozin, metformin and canagliflozin			657
	A10BK04, A10BD23	Ertugliflozin, metformin and ertugliflozin			40

a. Patients on treatment after propensity-score matched cohorts.

* Revoked on January 8, 2026

Abbreviations: OAD, oral antidiabetic medication, ATC, Anatomical Therapeutic Chemical, SGLT-2i, Sodium-glucose co-transporter 2 inhibitors, GLP-1 RA, Glucagon-like peptide-1 analogues, O, oral, P, parenteral, D, daily, W, weekly

Table S2: Adherence and persistence during 1-year follow-up (Original cohort).

	Original Cohort		
	**SGLT-2i**	**GLP-1 RA**	**p**
**N**	45102	7561	
Adherence % (95% CI)			
MPR (mean±SD)	92.10±12.43	87.14±20.83	0.001
Adherence prevalence	88.5 (88.21-88.79)	76.56 (75.61-77.51)	0.001
Adherence level			
None (<20)	0.21 (0.17-0.25)	0.21 (0.11-0.31)	
Poor (20-49)	1.85 (1.73-1.97)	1.9 (1.59-2.21)	0.001
Moderate (50-79)	9.44 (9.17-9.71)	21.32 (20.4-22.24)	
OR for adherence* (95% CI)	2.01 (1.89-2.15)	1 (Reference)	0.001
Persistence % (95% CI)			
3 months	88.68 (88.39-88.97)	87.91 (87.18-88.64)	0.050
6 months	80.68 (80.32-81.04)	79.3 (78.39-80.21)	0.005
12 months	70.14 (69.72-70.56)	66.51 (65.45-67.57)	0.001
Treatment persistence (days) (mean (95%CI))	299.44 (298.38-300.49)	294.16 (291.53-296.78)	0.001
HR for discontinuation** (95% CI)	0.94 (0.90–0.99)	1 (Reference)	0.011

*Logistic regression model (multivariate analysis)

**Cox proportional hazards model (multivariate analysis)

Abbreviations: PSM, propensity score-matched, SGLT-2i, Sodium-glucose co-transporter 2 inhibitors, GLP-1 RA, Glucagon-like peptide-1 analogues, CI, confidence interval; SD, standard deviation, OR, odds ratio, HR, hazard ratio

Table S3: Cox proportional hazards regression (after PSM) for discontinuation in patients with T2DM and cardiovascular disease (CVD) on sodium-glucose cotransporter-2 inhibitors (SGLT-2is) and glucagon-like peptide-1 receptor agonists (GLP-1 RAs).

	Univariate Analysis		Multivariate Analysis	
**Variables**	**HR (95% CI)**	**p**	**HR (95% CI)**	**p**
Cohort (SGLT2i)	0.67 (0.6-0.76)	0.001	0.66 (0.58-0.75)	0.001
**Sociodemographic characteristics**				
Sex (male)	0.88 (0.78-1.01)	0.057	0.93 (0.81-1.08)	0.318
Age	1 (0.99-1.01)	0.742	1 (0.99-1.01)	0.586
Healthcare area (Urban)	1.11 (0.98-1.25)	0.107	1.15 (0.95-1.3)	0.058
Socioeconomical level				
< 18000 €/year	1 (Reference)		1 (Reference)	
18000-100000 €/year	0.75 (0.37-1.52)	0.422	0.8 (0.4-1.61)	0.532
≥ 100000 €/year	0.89 (0.44-1.79)	0.748	0.93 (0.46-1.89)	0.84
**Clinical characteristics**				
Newly diagnosed T2DM (Yes)	1.09 (0.93-1.28)	0.311	1.2 (1.04-1.54)	0.035
Polypharmacy (Yes)	1.33 (0.6-2.94)	0.491	1.27 (0.56-2.86)	0.562
Monotherapy (Yes)	0.85 (0.63-1.15)	0.307	0.79 (0.58-1.08)	0.141
Insulin use (Yes)	1.18 (1.03-1.33)	0.012	1.18 (1.03-1.35)	0.016
**Cardiovascular disease**				
Atrial fibrillation (Yes)	0.86 (0.76-0.98)	0.032	0.88 (0.79-0.98)	0.033
Ischemic Heart Disease (Yes)	1.02 (0.9-1.15)	0.774	1.03 (0.87-1.2)	0.759
Heart failure (Yes)	1.14 (0.98-1.3)	0.060	1.18 (0.99-1.35)	0.063
Ischemic Stroke (Yes)	1.1 (0.92-1.3)	0.314	1.11 (0.89-1.37)	0.364
**Comorbidities**				
Hypertension (Yes)	0.93 (0.82-1.05)	0.279	0.91 (0.79-1.04)	0.158
Hyperlipidemia (Yes)	1 (0.88-1.12)	0.920	0.98 (0.86-1.11)	0.739
Chronic airways disease (Yes)	1.16 (1.02-1.33)	0.019	1.14 (1.02-1.3)	0.045
Anxiety (Yes)	1.23 (1.05-1.43)	0.008	1.19 (1.02-1.41)	0.029
Depression (Yes)	1.1 (0.91-1.33)	0.312	1.04 (0.86-1.27)	0.674
Cronic Kidney Disease (Yes)	1.25 (1.01-1.56)	0.044	1.23 (0.99-1.56)	0.061
Gout (Yes)	1.03 (0.74-1.43)	0.851	1.05 (0.76-1.47)	0.742
Obesity (Yes)	1.18 (0.85-1.67)	0.329	1.22 (0.86-1.69)	0.261

Abbreviations: SGLT2i, Sodium-glucose co-transporter 2 inhibitors, CI, confidence interval, HR, hazard ratio

Table S4: International Classification of Disease 10th edition (ICD-10) codes used to define the comorbidities in the study cohort [72].

**Disease**	**ICD-10 Codes**
Type 2 Diabetes Mellitus	E11
Anxiety	F40, F41
Atrial fibrillation	I48
Chronic kidney disease	I12, I13, N00, N01, N02, N03, N04, N05, N07, N11, N14, N17, N18, N19, Q61
Chronic respiratory disease	J41 - J44
Depression	F32, F33
Gout	M10, M1A
Heart failure	I11.0, I13.0, I13.2, I42.0, I50, I50.1, I50.9
Hyperlipidemia	E78
Hypertension	I10 - I16
Ischemic heart disease	I21 - I25
Ischemic stroke	I63, I64
Obesity	E66

Abbreviations: ICD, International Classification of Disease

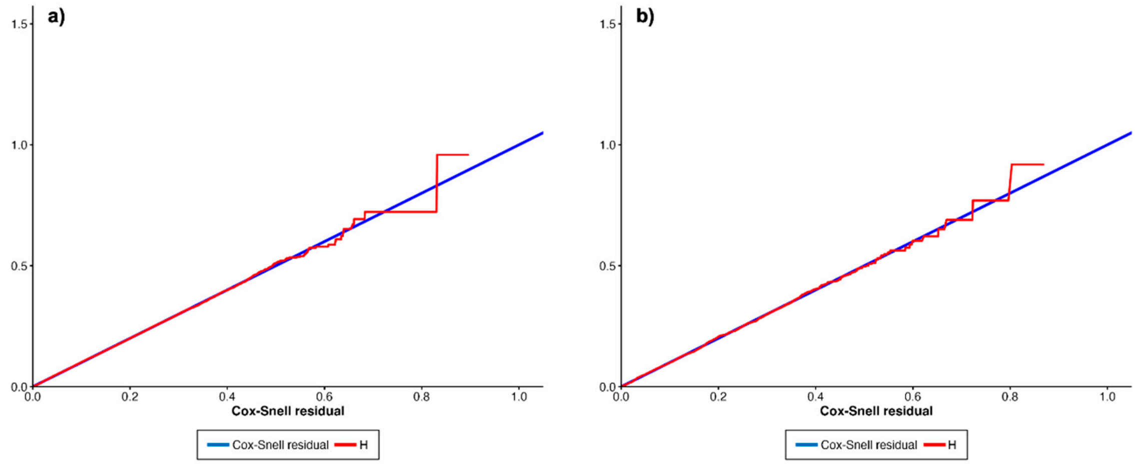

Figure S1. Cox-Snell residual plots on patients with T2DM on sodium-glucose cotransporter-2 inhibitors (SGLT-2is) and glucagon-like peptide-1 receptor agonists (GLP-1 RAs). a) All patients, b) Patients with cardiovascular disease.

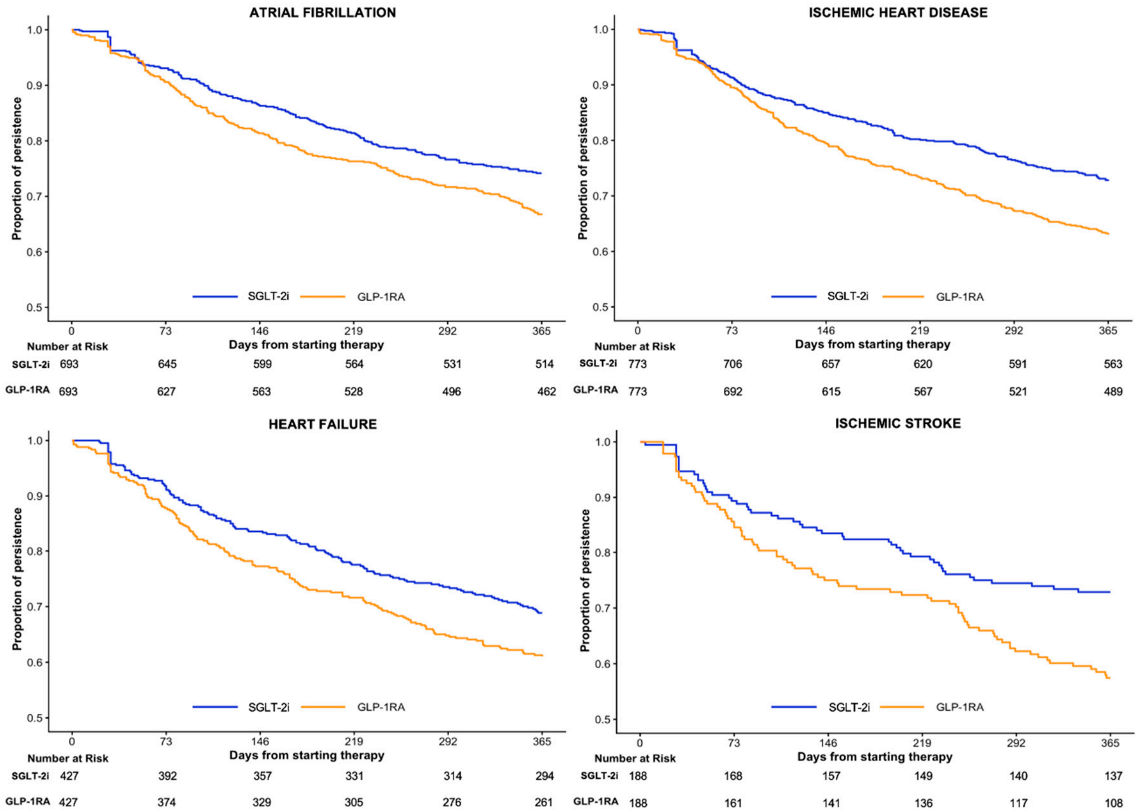

Figure S2. Kaplan-Meier analysis for treatment persistence by type cardiovascular disease (CVD) in patients on sodium-glucose cotransporter-2 inhibitors (SGLT-2is) and glucagon-like peptide-1 receptor agonists (GLP-1 RAs).

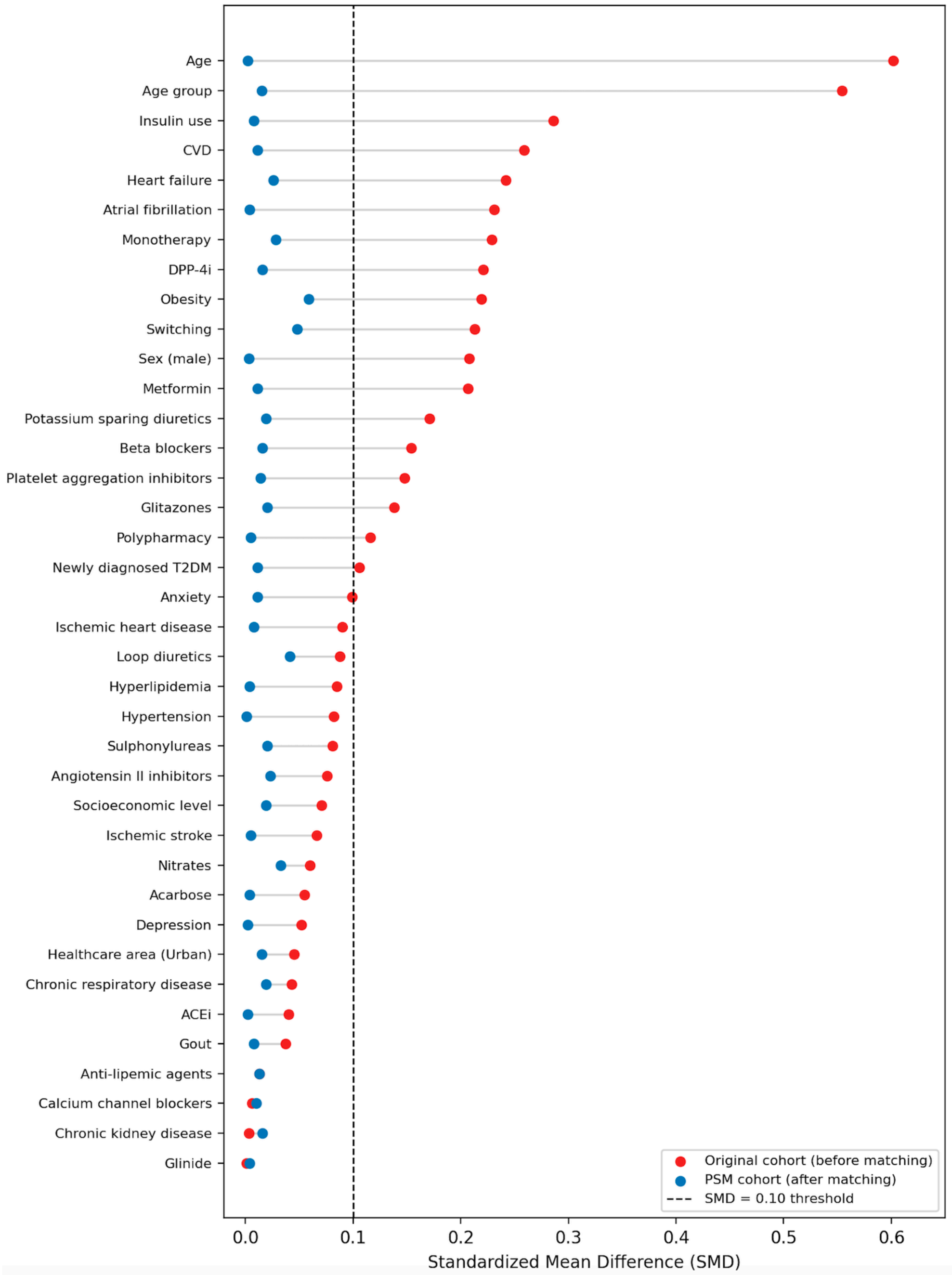

Figure S3: Standardized mean differences of baseline covariates before and after propensity score matching (Love Plot). (CVD, cardiovascular disease, DPP-4i, Dipeptidyl peptidase 4 inhibitors, T2DM, Type 2 Diabetes Mellitus, ACEi, Angiotensin converting enzyme inhibitors)
